# The Role of Perfusion Computed Tomography in the Prediction of Cerebral Hyperperfusion Syndrome

**DOI:** 10.1371/journal.pone.0019886

**Published:** 2011-05-20

**Authors:** Chien Hung Chang, Ting Yu Chang, Yeu Jhy Chang, Kuo Lun Huang, Shy Chyi Chin, Shan Jin Ryu, Tao Chieh Yang, Tsong Hai Lee

**Affiliations:** 1 Stroke Center and Department of Neurology, Chang Gung Memorial Hospital, Linkou Medical Center and College of Medicine, Chang Gung University, Kueishan, Taoyuan, Taiwan; 2 Department of Diagnostic Radiology, Chang Gung Memorial Hospital, Linkou Medical Center and College of Medicine, Chang Gung University, Kueishan, Taoyuan, Taiwan; 3 Department of Neurosurgery, Chang Gung Memorial Hospital, Linkou Medical Center and College of Medicine, Chang Gung University, Kueishan, Taoyuan, Taiwan; Cuban Neuroscience Center, Cuba

## Abstract

**Background:**

Hyperperfusion syndrome (HPS) following carotid angioplasty with stenting (CAS) is associated with significant morbidity and mortality. At present, there are no reliable parameters to predict HPS. The aim of this study was to clarify whether perfusion computed tomography (CT) is a feasible and reliable tool in predicting HPS after CAS.

**Methodology/Principal Findings:**

We performed a retrospective case-control study of 54 patients (11 HPS patients and 43 non-HPS) with unilateral severe stenosis of the carotid artery who underwent CAS. We compared the prevalence of vascular risk factors and perfusion CT parameters including regional cerebral blood volume (rCBV), regional cerebral blood flow (rCBF), and time to peak (TTP) within seven days prior to CAS. Demographic information, risk factors for atherosclerosis, and perfusion CT parameters were evaluated by multivariable logistic regression analysis. The rCBV index was calculated as [(ipsilateral rCBV - contralateral rCBV)/contralateral rCBV], and indices of rCBF and TTP were similarly calculated. We found that eleven patients had HPS, including five with intracranial hemorrhages (ICHs) of whom three died. After a comparison with non-HPS control subjects, independent predictors of HPS included the severity of ipsilateral carotid artery stenosis, 3-hour mean systolic blood pressure (3 h SBP) after CAS, pre-stenting rCBV index >0.15 and TTP index >0.22.

**Conclusions/Significance:**

The combination of severe ipsilateral carotid stenosis, 3 h SBP after CAS, rCBV index and TTP index provides a potential screening tool for predicting HPS in patients with unilateral carotid stenosis receiving CAS. In addition, adequate management of post-stenting blood pressure is the most important treatable factor in preventing HPS in these high risk patients.

## Introduction

Carotid angioplasty with stenting (CAS) is considered less invasive than carotid endarterectomy (CEA) and is an acceptable alternative treatment for carotid artery stenosis [1]–[3]. Major complications with CAS include cerebral embolism, vessel dissection, and hyperperfusion syndrome (HPS) [4]–[6]. In 1978, Spetzler et al. first described the phenomenon of hyperperfusion following surgical resection of arteriovenous malformations [7]. In 1981, Sundt et al. described this phenomenon in patients receiving carotid endarterectomy (CEA), in which a triad of signs and symptoms including ipsilateral throbbing headache, transient focal seizures, and intracranial hemorrhage defined HPS [8]. HPS often results in significant morbidity and mortality; therefore, identifying patients who are most at risk for HPS after CAS is crucial. [5], [6]

Although quantitative measurement of regional cerebral blood flow (rCBF) provided by positron emission tomography (PET) [9], [10] and xenon-enhanced computed tomography (CT) [11] can be considered the gold standard for assessment of cerebral perfusion, the availability of these imaging studies is limited. Perfusion CT has also been used to assess cerebral perfusion after brain infarct [9], [10]. The aim of this study was to clarify whether perfusion CT is a feasible and reliable tool in predicting HPS after CAS.

## Methods

### Objectives

The aim of this study was to determine if perfusion CT can reliably predict HPS after CAS. The degree of carotid artery stenosis was measured according to the methods of the North American Symptomatic Carotid Endarterectomy Trial (NASCET) [12], and a successful procedure was defined as a post-stent luminal narrowing of less than 30%. Patients with neurological deficits that related to the hemisphere perfused by the stenotic carotid artery (i.e., the ipsilateral side) were diagnosed with symptomatic carotid artery stenosis. Patients with ipsilateral throbbing headache, seizures, intracranial hemorrhages (ICH) and focal neurologic deficits after CAS were diagnosed with HPS [5], [8], [13]–[14]. Cerebral diffusion-weighted MRI was performed to rule out embolic stroke after CAS. In addition, 3-hour SBP (3 h SBP) after CAS was defined as the mean SBP within 3 hours after CAS.

### Participants

We retrospectively reviewed consecutive patients with unilaterally significant (>60%) carotid artery stenosis (both symptomatic and asymptomatic), who underwent CAS in Linkou Chang Gung Memory Hospital from April 2003 to September 2008. We conducted a matched case control study. Patients in the control group were age and sex matched. In addition, the degree of contralateral stenosis had to be below 50% with minimal difference (±10%) between two groups. Patients with HPS (case subjects) and patients without HPS (control subjects) were retrospectively recruited in the same period. A total of 54 subjects (11 HPS and 43 non-HPS) were included to the study, and all participants were interviewed and examined by one neurologist. All patients were informed about the best medical treatment and both invasive therapies (CEA and CAS), but all 54 patients decided to receive CAS.

Before CAS, the patients were pretreated with 100 mg/d of aspirin and 75 mg/d of clopidogrel for at least four days. In addition, all patients underwent perfusion CT within one week prior to the procedure. A neurologist evaluated all the patients before and after CAS to determine whether any patients showed neurological signs or symptoms. After CAS, the patients were transferred to the intensive care unit and BP was monitored hourly. The demographic information, atherosclerotic risk factors, associated conditions and indices of rCBV, rCBF and TTP on perfusion CT were collected for further analysis.

### Description of Procedures undertaken: Perfusion CT

**Figure 1:**
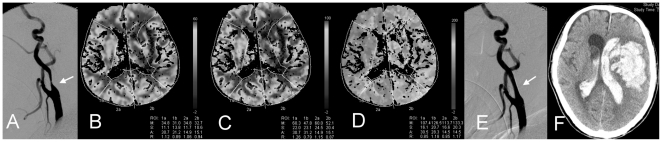
A 68-year-old man with a right hemispheric ischemic stroke. (**A**) Pre-stenting digital subtraction angiography reveals a 76% stenosis of the left internal carotid artery. A perfusion computed tomography (CT) study demonstrates a relatively decreased regional cerebral blood volume (rCBV) (**B**), decreased regional cerebral blood flow (rCBF) (**C**), and increased time to peak (TTP) (**D**) in the left hemisphere. The stenting procedure shows a 25% residual stenosis of the carotid artery (**E**). The rainbow on the right display reveals a range of rCBV from −2 to 60 ml/100 gm; rCBF from −2 to 100 ml/100 gm/min and TTP from −2 to 200 deciseconds. The patient suffered a sudden loss of consciousness two hours after stenting, and follow-up brain CT reveals a massive hematoma of the left basal ganglia, left frontal and temporal lobes with rupture into the ventricles (**F**).

Cerebral perfusion CT was performed on a CT unit equipped with a 16-detector array (SOMATOM Sensation 16, Siemens AG, Forchheim, Germany). After nonenhanced CT of the whole brain, two adjacent 12-mm-thick sections were selected at the level of the third ventricle and basal ganglia that covered the anterior, middle, and posterior cerebral artery territories. A bolus of 40 mL of nonionic iodinated contrast medium (350 mg/dL, Omnipaque™ (iohexol), GE healthcare, Ireland) was injected at a rate of 5 mL/s into an antecubital vein with a power injector. Five seconds after the injection, dynamic (continuous) scanning was initiated with the following technique: 80 kVp, 120 mA, four 24-mm-thick sections, and 0.5 seconds per rotation for 40 seconds. The time delay before the contrast material reached the brain parenchyma allowed for the acquisition of nonenhanced baseline images. The 5-mm-thick sections were reformatted into two 12-mm-thick sections. Therefore, each section was composed of 80 sequential images (40 prospective and 44 retrospective images). There were a total of 160 images (2 adjacent 12 mm sections) with a 0.5 second time resolution. For all scans, a 25 cm field of view was used, and scans were reconstructed with a matrix of 512*512 pixels. Relative values of regional cerebral blood flow (rCBF), regional cerebral blood volume (rCBV), and time to peak (TTP) (Figs. 1B, 1C, and 1D), based on the CT time attenuation curves for each pixel, were generated on the scanner's workstation using the software provided by the manufacturer (Syngo Somaris/5 VB 10B, Siemens Medical Solutions, Germany). The TTP index was calculated as [(ipsilateral TTP - contralateral TTP)/contralateral TTP]. The indices of rCBV and rCBF were also calculated in the same manner.

### Ethics

This study was conducted according to the principles expressed in the Declaration of Helsinki and was approved by the Institutional Review Board of Chang Gung Memorial Hospital. All patients provided written informed consent for the collection of samples and subsequent analysis.

### Statistical methods

Statistical analysis was performed using SPSS software for Windows version 16.0 (SPSS Inc, Chicago, IL). A two-tailed Student's t-test for continuous variables and chi-square test (or Fisher's exact test whenever appropriate) for discrete variables (Tables 1 and 2) were performed to compare the study characteristics associated with positive versus negative variables between HPS and non-HPS. To assess the ability of perfusion CT parameters to distinguish high-risk participants who actually had HPS from low-risk patients, we calculated the area under the receiver operating characteristic (AUROC) curve of CT perfusion parameters, which assessed the discrimination of the function. Sample size calculation for logistic regression is a complex problem, but Peduzzi [15] recommended that the smaller of the classes of the dependent variable have at least 10 events per parameter in the model. We calculated that we would obtain acceptable statistical power from this study if the sample size was greater than 50. Our total sample size was 54. Three multivariable logistic regression analyses were performed to identify which variables were independently associated with HPS. Our aim was not to develop a prediction model primarily based on preoperative clinical characteristics, but to assess whether a perfusion CT parameter was associated with HPS. Therefore, as is common in prediction research, we defined an independent association if the odds ratio (OR) had a value of p<0.05. Starting with a model that included preoperative clinical variables (based on their known or potential confounding effects on the relationship of HPS from the literature) [4], [5], [9], [10], [13] or initial univariate analyses with significant results (p<0.05), the insignificant (p>0.05) ones were excluded. This model was extended with the univariate significant TTP index. Finally, this last model was extended with the rCBV index to evaluate whether it further contributed to the clinical variables and TTP index in predicting HPS.

**Table 1:** Demographic data of patients with and without hyperperfusion syndrome.

	Without HPS (n = 43)	With HPS (n = 11)
Atherosclerotic risk factors, n (%)		
Age (years)	71.1 (4.6; 60–89)	71.5 (5.0; 81–95)
Gender (male)	39 (90.7)	10 (90.0)
Hypertension	34 (79.1)	8 (72.7)
Diabetes mellitus	13 (30.2)	4 (36.4)
Dyslipidemia	17 (39.5)	5 (45.5)
Smoking	23 (53.5)	6 (54.5)
TIA	6 (14.0)	1 (9.1)
Stroke	26 (60.5)	7 (63.6)
CAD	13 (30.2)	3 (27.3)
Carotid artery stenosis and procedure related		
Ipsilateral CA stenosis (%)	76.0 (8.4; 60–89)	85.4 (9.4; 81–95)*
Contralateral CA stenosis (%)	35.9 (3.7; 25–49)	42.4 (3.5; 30–49)
Residual CA stenosis (%)	19.6 (12.8; 0–30)	18.3 (6.9; 5–26)
Balloon pressure	9.8 (3.0; 6–18)	12.9 (3.4; 6–16)**
Pre-stent SBP	130.1 (12.9; 105–165)	131.8 (13.5; 109–149)
Post-stent 1 h SBP	132.3 (24.3; 87–183)	146.3 (18.1; 121–166)
Post-stent 3 h SBP	132.1 (22.4; 77–187)	169.7 (22.6; 130–208)**
Post-stent 6 h SBP	130.3 (20.9; 81–183)	139.1 (26.9; 104–198)*
Post-stent 24 h SBP	126.7 (14.6; 108–153)	129.6 (13.8; 102–142)

Continuous data are displayed as mean (SD; range) and discrete data are presented as count (%).

*p<0.05;

**p<0.001;

HPS: hyperperfusion syndrome; TIA: transient ischemic attack; CAD: coronary artery disease, CA: carotid artery; SBP: systolic blood pressure.

**Table 2:** Comparison of perfusion CT parameters between patients with and without hyperperfusion syndrome.

	Without HPS (n = 43)	With HPS (n = 11)
**Perfusion CT parameters**		
rCBV index (%)	0.04 (0.71)	0.16 (0.08)**
rCBF index (%)	0.04 (0.14)	−0.96 (0.2)
TTP index (%)	0.06 (0.23)	0.29 (0.37)**

Continuous data are displayed as mean (SD); p<0.05;

**p<0.001;

CT: computed tomography; HPS: hyperperfusion syndrome; rCBF index: regional cerebral blood flow index = [(ipsilateral rCBF−contralateral rCBF)/contralateral rCBF]; rCBV index: regional cerebral blood volume index = [(ipsilateral rCBV−contralateral rCBV)/contralateral rCBV]; TTP index: time to peak index = [(ipsilateral TTP−contralateral TTP)/contralateral TTP].

## Results

A total of 54 patients (11 HPS patients and 43 non-HPS) with unilateral severe stenosis of the carotid artery underwent CAS. The distribution of demographic data, clinical characteristics and outcomes are shown in Table 1. Among the 11 patients with HPS, three died due to massive ICH after CAS. Patients in the HPS group had a greater severity of carotid artery stenosis, larger balloon pressure and higher 3 h SBP after CAS (p<0.05, Table 1) compared to the non-HPS group. There were no significant differences in the distribution of atherosclerotic risk factors or clinical status between patients with and without HPS.

Imaging analysis was performed on all 54 patients using regions of interest (ROIs) drawn on the perfusion CT scans by an experienced neuroradiologist (S.C.C.). An ROI was drawn on each hemisphere in each of the 54 patients for a total of 108 ROIs generated from the ipsilateral middle cerebral artery (MCA) territory and 108 ROIs from the contralateral MCA territory. The mean values of the perfusion parameters from the ROIs are shown in Table 2. While the mean rCBV and mean TTP were both significantly higher in the HPS patients compared to the non-HPS patients (all p<0.05), there was no significant difference between the two groups with regards to rCBF.

**Figure 2:**
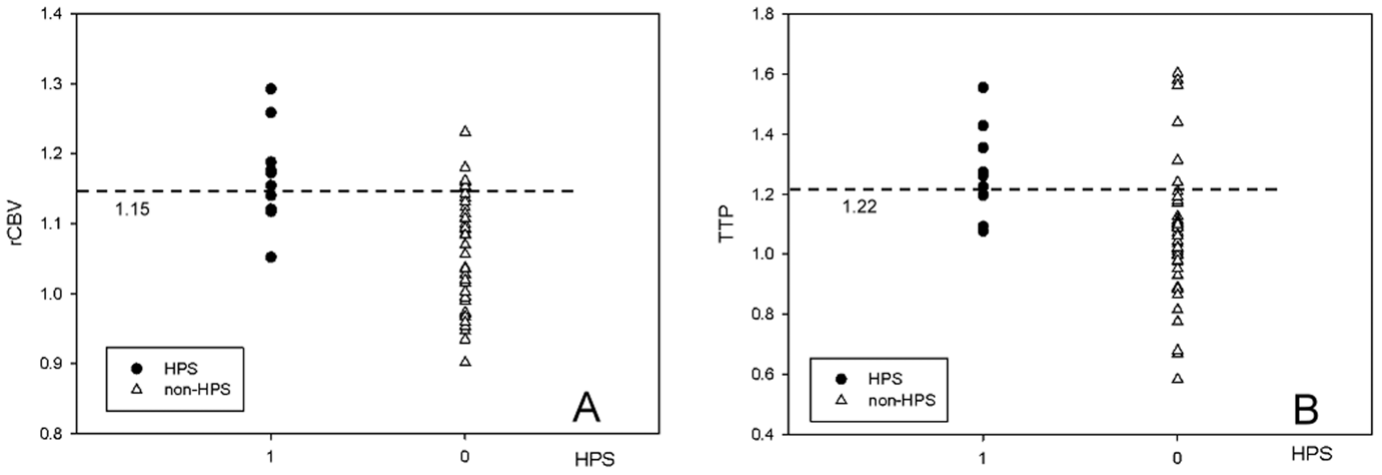
rCBV (A) and TTP (B) in HPS and non-HPS patients. From univariate discrimination analysis by receiver operating characteristic (ROC) curves, the threshold levels for the best discrimination of HPS and non-HPS are 1.15 and 1.22 for rCBV and TTP, respectively.

From the discrimination analysis based on the receiver operating characteristic (AUROC) curve, the thresholds for the discrimination of HPS and non-HPS were estimated to be approximately 0.15 for the rCBV index (AUROC = 0.74; confidence interval (CI) = 0.71–0.93) and 0.22 for the TTP index (AUROC = 0.65, CI = 0.62–0.91) if each parameter was used as a single discriminator (Figures 2A and 2B). Furthermore, the rCBV index tended to be more efficient in predicting HPS compared to the TTP index, although this difference did not reach statistical significance.

Stepwise multivariable logistic regression models (Table 3) for predicting HPS were devised to assess the incremental contribution of perfusion parameters to the clinical evaluation. For HPS, ipsilateral carotid stenosis and 3 h SBP after CAS were used for the clinical model (chi-square = 19.4, p = 0.03). The addition of the TTP index increased the power of the model (increment of chi-square = 4.12, df = 1, p = 0.03). The addition of the rCBV index further increased the power of the model (increment of chi-square = 3.07, df = 1, p = 0.04), even though the TTP index was not significantly associated with HPS.

**Table 3:** Multivariable logistic regression analysis of hyperperfusion syndrome after carotid stenting.

	OR (95% CI)		
	Model 1Clinical characteristics	Model 2Clinical characteristics+ TTP index	Model 3Clinical characteristics+ TTP index+ rCBV index
Clinical characteristics			
Age	1.13 (0.93–1.43)	1.18 (0.9–1.56)	1.18 (0.9–1.56)
Hypertension	0.39 (0.05–3.09)	0.15 (0.01–1.68)	0.15 (0.01–1.68)
Ipsilateral stenosis	1.25 (1.04–1.5)*	1.24 (1.03–1.49)*	1.23 (1.01–1.50)*
Contralateral stenosis	1.03 (0.99–1.05)	1.02 (0.99–1.06)	1.02 (0.99–1.06)
Balloon pressure	1.12 (0.81–1.56)	1.24 (0.86–1.77)	1.24 (0.86–1.77)
3 h SBP after CAS	2.18 (1.3–3.56)*	1.45 (1.08–1.93)*	1.45 (1.37–1.53)*
Perfusion CT parameters			
TTP index>0.22	----------	4.27 (1.11–47.84)*	3.44 (0.89–53.36)
rCBV index>0.15	----------	----------	6.89 (1.08–43.91)*
ΔX^2^	----------	4.12 (df = 1)*from Model 1	3.07 (df = 1)*from Model 2
AUROC (95%CI)	0.64 (1.12–2.87)	0.72 (1.24–2.89)	0.74 (0.64–0.89)

*p<0.05;

**p<0.001;

OR: odds ratio, CI: confidence interval, 3 h SBP after CAS: mean systolic blood pressure after carotid artery stenting within 3 hours, TTP index: time to peak index = [(ipsilateral TTP−contralateral TTP)/contralateral TTP], rCBV index: regional cerebral blood volume index = [(ipsilateral rCBV−contralateral rCBV)/contralateral rCBV], Δx2 (increment of chi-square) at steps 2 and 3 represent the statistical significance of any improvement in prediction as a result of that variable being entered into the model, AUROC: area under the receiver operating characteristic curve.

## Discussion

To the best of our knowledge, this is first time HPS has been characterized in a subgroup of patients with unilateral severe carotid artery stenosis using a combination of clinical characteristics and perfusion CT parameters. The independent patient characteristics were ipsilateral carotid artery stenosis, balloon pressure and the 3 h SBP after CAS. The present study also evaluated whether the TTP index and rCBV index were independently associated with HPS after CAS. As shown in Table 3, we found that Model 3, which included a TTP index >0.22 and an rCBV index >0.15, was statistically superior to the other two models in predicting HPS in our patients. These associations persisted after including the preoperative patient characteristics in addition to a TTP index >0.22. These results suggest that a model that includes the clinical characteristics, a TTP index >0.22 and an rCBV index >0.15 may be useful in the predicting HPS in patients with unilateral carotid stenosis undergoing CAS.

The potential HPS risk factors following CEA include age, hypertension, severity of ipsilateral carotid stenosis, and presence of contralateral stenosis or occlusion [4], [6], [12], [13], [16]–[18]. Owing to the study design and hypothesis, we controlled for age, gender and contralateral carotid artery stenosis <50%. In our retrospective study, only the severity of ipsilateral carotid artery stenosis, balloon pressure and the 3 h SBP after CAS were significant when comparing the non-HPS and HPS groups. High blood pressure is known to be a significant risk factor for HPS after carotid intervention [16], [19]–[20]. The difference between systemic blood pressure and venous back pressure determines cerebral perfusion pressure [17]. Reperfused blood flow in a severely hypoperfused vascular bed leads to profound vasodilatation and increased permeability, which can be augmented by increased BP [16]. Therefore, an ominous elevation of BP may increase cerebral perfusion pressure and predispose to ICH during reperfusion after carotid recanalization. Our patients who had a higher 3 h SBP after CAS had a significantly increased incidence of HPS compared to those with a lower SBP (p<0.05, Tables 1 and 3). Therefore, in high risk patients, a more aggressive control of SBP after CAS should decrease the incidence of HPS.

Cerebral perfusion can be measured by various tools including PET [9], [10], magnetic resonance imaging [21], single photon emission computed tomography [22] and xenon CT [11]. Of these techniques, PET is the current standard of reference for assessing cerebral blood flow and brain metabolism [9], [10]. Recently, perfusion CT has been shown to correlate well with the PET data. Previous investigations have also indicated that perfusion CT parameters such as TTP, rCBV, and rCBF are useful both for the delineation of hypoperfused brain tissue in acute stroke and for predicting outcome [23]. In recent years, perfusion CT has also been used to evaluate chronic ischemia associated with hemodynamic compromise resulting from high grade carotid stenosis [24], [25]. However, although perfusion CT can obtain quantitative data regarding brain perfusion, previous studies [26] have shown that absolute measurements are unable to provide accurate results due to limitations of the underlying model. In addition, the choice of arterial input function (AIF) and selection of ROIs are operator dependent [27], affecting consistency among different investigators. To overcome this pitfall, our perfusion parameters were semi-quantitatively corrected, by using as a reference, data from the mirror image regions in the contralateral hemisphere [28]. In the current study, we also used semi-quantitative indices of TTP, rCBF and rCBV as the basis of comparison between the two hemispheres, with the assumption that the perfusion of the contralateral hemisphere was normal. For this reason we focused only on patients with unilateral carotid stenosis.

Even with severe carotid stenosis or occlusion, distal brain perfusion and rCBF may remain normal or be only mildly decreased, with or without increasing rCBV, if collateral circulation is adequate [29]. TTP can indicate the local arrival of the contrast bolus, and in particular, depict the location and extent of ischemia [30], [31]. Blood flow via collateral routes may take a longer time to reach the brain than via the more normal, direct arterial routes, thus leading to delays in TTP [29]. According to the mathematical relationship, TTP = rCBV/rCBF, the synergistic effect of decreased rCBF and increased rCBV will result in a large change in TTP value. Furthermore, TTP may be more sensitive than rCBV or rCBF in detecting the presence of altered cerebral hemodynamics, especially in acute stroke and cerebrovascular occlusive disease [32], [33]. Our findings support previous reports [34], [35] and suggest a model that can predict the development of HPS using clinical characteristics and TTP index. In addition, this model is statistically superior in performance to other models that are based on clinical variables alone.

We also found that there was a significant increase in TTP as the rCBV index was added to Model 3, even if the TTP index was statistically insignificant. These results were substantiated by a recent report by Trojanowska et al. [36]. Similarly, in a study by Tseng et al. [37] using GE perfusion CT workstation those patients who developed HPS after CAS showed a prolonged dMTT of more than 3 seconds. These patients also showed a tendency of prolonged rCBV, though statistically non-significant. In other words, even though TTP index prolongation was remarkable, the rCBV index increased at the same time. This finding suggests a failure of autoregulation which results in maximal dilation of cerebral arterioles over time, with subsequent loss of the ability of the arteriole to constrict when normal perfusion pressure is restored. Under such conditions, luxury perfusion after successful CAS may lead to circulatory overload followed by hyperperfusional injury and the potential development of HPS. Consequently, those patients with increased rCBV and TTP are at higher risk of developing HPS, compared to groups with normal rCBV values.

### Limitations

Our study had several limitations. First, since contrast media must be administered as a bolus, imaging can be performed only once per imaging session in a first-pass bolus study. Although the quality of an intravenous bolus depends on many physiologic parameters, a flow rate of 6–10 mL/s appears sufficient to obtain a rather narrow distribution of TTP values in the healthy hemisphere [38]. Because we were able to achieve a flow rate of only 5 mL/s in our patients, our TTP values had a broad range. We were also limited, using perfusion CT, to single-section measurements. Ideally, a multi-section acquisition that covers a larger volume of the brain would be desirable. Although a single section technique is capable of localizing the infarction, it is not able to show the extent of the infarction in all three dimensions. This does not necessarily imply, however, that perfusion CT is meaningless in cases of chronic ischemia with hemodynamic compromise. A future perfusion CT study with a larger number of patients is warranted to confirm our findings in cases of chronic ischemia with hemodynamic compromise. Finally, the small case number and low incidence of HPS in our study limits its application. Larger prospective studies of CAS are necessary to confirm our results.

In conclusion, the combination of clinical characteristics coupled with rCBV and TTP indices are a potential screening tool in the pre-stent evaluation for HPS in patients with unilateral severe carotid artery stenosis. In addition, adequate management of post-stenting BP is the most important treatable factor in preventing HPS in high risk patients.

## References

[1] Golledge J, Mitchell A, Greenhalgh RM, Davies AH (2000). Systematic comparison of the early outcome of angioplasty and endarterectomy for symptomatic carotid artery disease.. Stroke.

[2] Hobson RW, Brott T, Ferguson R, Roubin G, Moore W (1997). Crest: Carotid revascularization endarterectomy versus stent trial.. Cardiovasc Surg.

[3] Malek AM, Higashida RT, Phatouros CC, Lempert TE, Meyers PM (2000). Stent angioplasty for cervical carotid artery stenosis in high-risk symptomatic nascet-ineligible patients.. Stroke.

[4] McCabe DJ, Brown MM, Clifton A (1999). Fatal cerebral reperfusion hemorrhage after carotid stenting.. Stroke.

[5] Meyers PM, Higashida RT, Phatouros CC, Malek AM, Lempert TE (2000). Cerebral hyperperfusion syndrome after percutaneous transluminal stenting of the craniocervical arteries.. Neurosurgery.

[6] Morrish W, Grahovac S, Douen A, Cheung G, Hu W (2000). Intracranial hemorrhage after stenting and angioplasty of extracranial carotid stenosis.. AJNR.

[7] Spetzler RF, Wilson CB, Weinstein P, Mehdorn M, Townsend J (1978). Normal perfusion pressure breakthrough theory.. Clin Neurosurg.

[8] Sundt TM, Sharbrough FW, Piepgras DG, Kearns TP, Messick JM (1981). Correlation of cerebral blood flow and electroencephalographic changes during carotid endarterectomy: With results of surgery and hemodynamics of cerebral ischemia.. Mayo Clin Proc.

[9] Gillard JH, Minhas PS, Hayball MP, Bearcroft PW, Antoun NM (2000). Assessment of quantitative computed tomographic cerebral perfusion imaging with h2(15)o positron emission tomography.. Neurol Res.

[10] Kudo K, Terae S, Katoh C, Oka M, Shiga T (2003). Quantitative cerebral blood flow measurement with dynamic perfusion ct using the vascular-pixel elimination method: Comparison with h2(15)o positron emission tomography.. AJNR.

[11] Firlik AD, Kaufmann AM, Wechsler LR, Firlik KS, Fukui MB (1997). Quantitative cerebral blood flow determinations in acute ischemic stroke. Relationship to computed tomography and angiography.. Stroke.

[12] Ferguson GG, Eliasziw M, Barr HW, Clagett GP, Barnes RW (1990). The north american symptomatic carotid endarterectomy trial: Surgical results in 1415 patients.. Stroke.

[13] Abou-Chebl A, Yadav JS, Reginelli JP, Bajzer C, Bhatt D (2004). Intracranial hemorrhage and hyperperfusion syndrome following carotid artery stenting: Risk factors, prevention, and treatment.. J Am Coll Cardiol.

[14] Bernstein M, Fleming JF, Deck JH (1984). Cerebral hyperperfusion after carotid endarterectomy: A cause of cerebral hemorrhage.. Neurosurgery.

[15] Peduzzi P, Concato J, Kemper E, Holford TR, Feinstein AR (1996). A simulation study of the number of events per variable in logistic regression analysis.. J Clin Epidemiol.

[16] Howell M, Krajcer Z, Dougherty K, Strickman N, Skolkin M (2002). Correlation of periprocedural systolic blood pressure changes with neurological events in high-risk carotid stent patients.. J Endovasc Ther.

[17] Markus HS (2004). Cerebral perfusion and stroke.. J Neurol Neurosurg Psychiatry.

[18] Qureshi AI, Saad M, Zaidat OO, Suarez JI, Alexander MJ (2002). Intracerebral hemorrhages associated with neurointerventional procedures using a combination of antithrombotic agents including abciximab.. Stroke.

[19] Caplan LR, Skillman J, Ojemann R, Fields WS (1978). Intracerebral hemorrhage following carotid endarterectomy: A hypertensive complication?. Stroke.

[20] Ouriel K, Shortell CK, Illig KA, Greenberg RK, Green RM (1999). Intracerebral hemorrhage after carotid endarterectomy: Incidence, contribution to neurologic morbidity, and predictive factors.. J Vasc Surg.

[21] Ernst T, Chang L, Itti L, Speck O (1999). Correlation of regional cerebral blood flow from perfusion mri and spect in normal subjects.. Magn Reson Imaging.

[22] Sakai F, Nakazawa K, Tazaki Y, Ishii K, Hino H (1985). Regional cerebral blood volume and hematocrit measured in normal human volunteers by single-photon emission computed tomography.. J Cereb Blood Flow Metab.

[23] Tomandl BF, Klotz E, Handschu R, Stemper B, Reinhardt F (2003). Comprehensive imaging of ischemic stroke with multisection ct.. Radiographics.

[24] Jain R, Hoeffner EG, Deveikis JP, Harrigan MR, Thompson BG (2004). Carotid perfusion ct with balloon occlusion and acetazolamide challenge test: Feasibility.. Radiology.

[25] Roberts HC, Dillon WP, Smith WS (2000). Dynamic ct perfusion to assess the effect of carotid revascularization in chronic cerebral ischemia.. AJNR.

[26] Klotz E, Konig M (1999). Perfusion measurements of the brain: Using dynamic ct for the quantitative assessment of cerebral ischemia in acute stroke.. Eur J Radiol.

[27] Sanelli PC, Lev MH, Eastwood JD, Gonzalez RG, Lee TY (2004). The effect of varying user-selected input parameters on quantitative values in ct perfusion maps.. Acad Radiol.

[28] Koenig M, Kraus M, Theek C, Klotz E, Gehlen W (2001). Quantitative assessment of the ischemic brain by means of perfusion-related parameters derived from perfusion ct.. Stroke.

[29] Lu J, Li KC, Hua Y (2005). Primary study on imaging in transient ischemic attacks.. Chin Med J (Engl).

[30] Gibbs JM, Wise RJ, Leenders KL, Jones T (1981). Evaluation of cerebral perfusion reserve in patients with carotid-artery occlusion.. Lancet.

[31] Norrving B, Nilsson B, Risberg J (1982). Rcbf in patients with carotid occlusion. Resting and hypercapnic flow related to collateral pattern.. Stroke.

[32] Kluytmans M, van der Grond J, Viergever MA (1998). Gray matter and white matter perfusion imaging in patients with severe carotid artery lesions.. Radiology.

[33] Neumann-Haefelin T, Wittsack HJ, Wenserski F, Siebler M, Seitz RJ (1999). Diffusion- and perfusion-weighted MRI. The DWI/PWI mismatch region in acute stroke.. Stroke.

[34] Soinne L, Helenius J, Tatlisumak T, Saimanen E, Salonen O (2003). Cerebral hemodynamics in asymptomatic and symptomatic patients with high-grade carotid stenosis undergoing carotid endarterectomy.. Stroke.

[35] Waaijer A, van Leeuwen MS, van Osch MJ, van der Worp BH, Moll FL (2007). Changes in cerebral perfusion after revascularization of symptomatic carotid artery stenosis: Ct measurement.. Radiology.

[36] Trojanowska A, Drop A, Jargiello T, Wojczal J, Szczerbo-Trojanowska M (2006). Changes in cerebral hemodynamics after carotid stenting: Evaluation with ct perfusion studies.. J Neuroradiol.

[37] Tseng YC, Hsu HL, Lee TH, Hsieh IC, Chen CJ (2009). Prediction of cerebral hyperperfusion syndrome after carotid stenting: A cerebral perfusion computed tomography study.. J Comput Assist Tomogr.

[38] Reichenbach JR, Rother J, Jonetz-Mentzel L, Herzau M, Fiala A (1999). Acute stroke evaluated by time-to-peak mapping during initial and early follow-up perfusion ct studies.. AJNR.

